# On the Physiology of the Brain and Nerves

**Published:** 1800-10

**Authors:** T. Purton

**Affiliations:** Alcester


### *On the Physiology of the Brain and Nerves.*

To the Editors of the Medical and Physical Journal.

Gentlemen,

If you will do me the favour to admit into your valuable Journal the following Remarks on the Physiology of the Nerves, which have been drawn up with a diffidence commensurate with the acknowledged difficulty attending this interesting subject; and if it should be the means of furnishing any hint which, matured by some abler hand, may lead to a more clear and satisfactory investigation of that wonderful part of the animal œconomy, I shall feel myself highly gratified.

Many Physiologists have endeavoured to discover, whether sensation and motion are produced by vibration along the course of the nerve, or by a fluid; their researches have generally proved unsatisfactory, for want of even a probable elucidation of the fact.

Although this is a part of the physiology of the animal œconomy, which may not admit of absolute proof; still, I think, there may be a few facts advanced, which might, in the course of time, be the means of rendering this intricate subject more clear. Those facts which we do know, namely, that the nerves are the principal agents by which sensation and motion are produced, and that the cause which generally sets them in action rests in the brain, are so clear and demonstrative, that there cannot be two opinions on the matter; as also that, if a nerve be divided, the part to which it is distributed is immediately deprived of the faculty of feeling and motion. It is the means by which this is brought about that still lies in a labyrinth of mystery, and probably ever will. But let the difficulty be ever so great, it should not deter any one who has something new to offer, from contributing his mite; as by the union and investigation of several data, the truth may at last be discovered.—Upon this principle I take the liberty of submitting the following observations.

Many eminent Physiologists have declared, that the opinion of the animal spirits being a fluid, and that this fluid is conveyed from the brain by the nerves to the different parts of the body for the purpose of giving sensation and action, must undoubtedly be ill founded, as a fluid has never yet been discovered in the nerves. So far it may be true, that the nerves do not convey any thing which is perceptible, or that possesses altogether together the properties of a fluid; ſtill they may convey, as conductors, that ſubtle matter which has got the name of the electrical fluid.

Why may not this penetrating fluid, which pervades all bodies, be the primum mobile, or grand cauſe, which produces the wonderful phænomena in the nervous ſyſtem? Why may not the nerves be the ſole diſtributors of this matter, however produced, and muſcular action, if not ſenſation, depend entirely upon its influence?

That muſcular motion may be cauſed by the action of the electrical fluid, the experiments of Galvani and Valli, the properties of the torpedo and other electric fiſh, ſufficiently demonſtrate. It is worthy of remark, and much to our preſent purpoſe, that in thoſe fiſh which have the peculiar power at will of electrifying any ſubſtance within their reach, that their bodies are little elſe but a bundle of nerves, by far a greater proportion than any other animal whatever; and the nerves are likewiſe arranged in a line with the part which gives the ſtroke: a ſtrong evidence this in ſupport of the opinion now advanced; or why ſhould Nature make uſe of the ſubſtance called nerve, in preference to any other, as the beſt medium for collecting and diſcharging the electrical fluid?

The velocity with which impreſſions are communicated to and from the brain, cannot well be produced by any other known means, but by ſome *peculiar modification* of the ſame principle upon which the effects of electricity depend.

Another corroborating teſtimony of this opinion may be deduced from the effects that a certain ſtate of the atmoſphere produces on all, and particularly ſome, conſtitutions. From experiments which have been made, it appears that the circumambient air at thoſe times is unfavourable for electric inveſtigation, and evidently diminiſhes the effect, and perhaps the quantum, of this very ſubtle and active matter. Its preſence, then, is here proved to be highly neceſſary for the continuance of life, as the leaſt diminution in quantity cauſes ſuch conſiderable derangement in the animal functions.

If it could be once proved that a *principal* property of the brain was for the purpoſe of collecting, by the medium of the circulating fluids, the electric matter, and diſtributing the ſame by the nerves to all parts of the body, it might probably throw ſome light on the uſe of the ganglions. Theſe ſwellings differ from nerve in being more vaſcular, and likewiſe more pulpy, reſembling in their ſtructure the ſubſtance of the brain. It may not, therefore, be a very unreaſonable idea, to conſider them as ſo many little brains, aſſiſting the great maſs, by retaining and diftributing this ufeful fluid as the body may require it.

I have often remarked that the ganglions are diftributed in greater numbers, and alfo are in fize much larger, in the courfe of the great fympathetic nerves, termed by Anatomifts the par vagum and intercoftal. As thefe are the moft remarkable nerves in the body, whofe branches are diftributed very remote from the great brain, there might reafonably be wanted fome other fubordinate apparatus than merely nerve, not only to generate, but alfo to retain a fufficient quantity of this circulating fluid.

Dr. Monro's cafe of the child born without a head, certainly ftrengthens, and that in a very great degree, the before-mentioned opinion. It proves, that mufcular action can even exift independent of a brain; and that the caufe which produces nervous energy, muft neceffarily be derived from fome other fource.

The ufe of the nerves, under this idea, appears to be, *principally*, as fo many quick conductors, conveying fenfation and motion to every part of the body. The brain and ganglions may alfo be compared; the firft, to a large refervoir for the electric fluid; and the latter, to fo many knobs, anfwering fimilar intentions in the grand machine of Nature as thofe do which are ufed for experimental purpofes in electricity.

I am fully aware that there may be fome and even many objections advanced, in oppofition to this opinion; but I flatter myfelf that they will not prove fo conclufive and fatisfactory as thofe which have been already oppofed to the doctrine of vibrations.

Thus have I ventured to give my thoughts upon a fubject of great importance; and however they may be received in other refpects, I hope my claim for good intention will not be difallowed.

I am, your's, refpectfully,

*Akester, Aug.* 14, 1800. T. PURTON.

